# Cross-sectional evaluation of pharmaceutical care competences in nurse education: how well do curricula prepare students of different educational levels?

**DOI:** 10.1186/s12912-023-01646-6

**Published:** 2024-02-06

**Authors:** Elyne De Baetselier, Nienke E. Dijkstra, Luis M. Batalha, Paulo A. Carvalho Ferreira, Izabela Filov, Vigdis A. Grøndahl, Jana Heczkova, Ann K. Helgesen, Sue Jordan, Igor Karnjuš, Petros Kolovos, Gero Langer, Manuel Lillo-Crespo, Alba Malara, Hana Padyšaková, Mirko Prosen, Dorina Pusztai, Bence Raposa, Jorge Riquelme-Galindo, Jana Rottková, Carolien G. M. Sino, Francesco Talarico, Nicola Tingle, Styliani Tziaferi, Bart Van Rompaey, Tinne Dilles

**Affiliations:** 1 Faculty of Medicine and Health Sciences, University of Antwerp, Antwerp, Belgium; 2 University of Applied Sciences Utrecht, Research Group Care for the Chronically Ill, Utrecht, Netherlands; 3 Higher School of Nursing of Coimbra, Health Sciences Research Unit: Nursing, Coimbra, Portugal; 4 University “St.Kliment Ohridski”, Bitola, Republic of North Macedonia; 5 Østfold University College, Faculty of Health and Welfare, Halden, Norway; 6 Institute of Nursing Theory and Practice, First Faculty of Medicine, Charles University, Prague, Czech Republic; 7 Department of Nursing, Swansea University, Swansea, Wales, UK; 8 Department of Nursing, Faculty of Health Sciences, University of Primorska, Izola, Slovenia; 9 Department of Nursing, Laboratory of Integrated Health Care, University of Peloponnese, Tripolis, Greece; 10 Medical Faculty, Institute of Health and Nursing Sciences, Martin-Luther-Universitat Halle-Wittenberg, Halle (Saale), Germany; 11 Department of Nursing, Alicante University, Alicante, Spain; 12 ANASTE-Humanitas Foundation, Rome, Italy; 13 Faculty of Nursing and Professional Health Studies, Slovak Medical University in Bratislava, Bratislava, Slovakia; 14 Institute of Nursing Sciences, Basic Health Sciences and Health Visiting, University of Pecs Faculty of Health Sciences, Pecs, Hungary

**Keywords:** Nursing, Competences, Knowledge, Skills, Attitudes, Education, Pharmaceutical care

## Abstract

**Background:**

Nurses play an important role in interprofessional pharmaceutical care. Curricula related to pharmaceutical care, however, vary a lot. Mapping the presence of pharmaceutical care related domains and competences in nurse educational programs can lead to a better understanding of the extent to which curricula fit expectations of the labour market. The aim of this study was to describe 1) the presence of pharmaceutical care oriented content in nursing curricula at different educational levels and 2) nursing students’ perceived readiness to provide nurse pharmaceutical care in practice.

**Methods:**

A quantitative cross-sectional survey design was used. Nursing schools in 14 European countries offering educational programs for levels 4–7 students were approached between January and April 2021. Through an online survey final year students had to indicate to what extent pharmaceutical care topics were present in their curriculum.

**Results:**

A total of 1807 students participated, of whom 8% had level 4–5, 80% level 6, 12% level 7. Up to 84% of the students indicated that pharmaceutical care content was insufficiently addressed in their curriculum. On average 14% [range 0–30] felt sufficiently prepared to achieve the required pharmaceutical care competences in practice. In level 5 curricula more pharmaceutical care domains were absent compared with other levels.

**Conclusions:**

Although several pharmaceutical care related courses are present in current curricula of level 4–7 nurses, its embedding should be extended. Too many students perceive an insufficient preparation to achieve pharmaceutical care competences required in practice. Existing gaps in pharmaceutical care should be addressed to offer more thoroughly prepared nurses to the labour market.

**Supplementary Information:**

The online version contains supplementary material available at 10.1186/s12912-023-01646-6.

## Background

Healthcare practice is constantly changing. Many countries are trying to improve healthcare by revising the roles of health professionals, including nurses. The evolution of nurses’ roles brings with it new competencies and broadness in caring for patients. For example, in an increasing number of European countries, nurses can independently prescribe medications for selected patients. Until the 1990’s, in mainstream practice, prescribing was largely restricted to physicians [1]. Targeted education programs prepare nursing students to take on existing and new roles in clinical practice. Education is an important opportunity for nurses to expand and accelerate the acquisition of fundamental knowledge, skills, and behaviours and, in particular, to recognize their roles and responsibilities related to quality of care. Despite several reforms in European nurse education over the past two decades, curricula still vary considerably [2]. The same trend is expected for nurse educational programs on pharmaceutical care. In this study, pharmaceutical care is defined as ‘the contribution of pharmacists and other health professionals to the care of individuals in order to optimise the use of medicines and improve health outcomes’.This definition is based on the definitions of Hepler and Strand (1990) [3] and the Council of Europe (2020) [4], where the interprofessional dimension of pharmaceutical care is pointed out. Every day, millions of nurses worldwide contribute to the care of patients, almost all of whom are treated with medications. Nurse educators are expected to prepare nurse students for practise and provide them with the competencies needed to provide safe, high-quality pharmaceutical care. Because of the changing roles in clinical practice, it is advisable to regularly evaluate the fit between labour market expectations and the content of nurse educational programs.

Recently, the NuPhaC-EU framework was developed and evaluated to provide more role transparency [5]. Nurses have responsibilities in different pharmaceutical care domains, such as administration of medicines, management of medicines adverse effects, medicines adherence, and medication safety. Within these responsibilities, an extensive list of activities has been described. Between and within European countries these responsibilities differ in terms of what nurses are allowed to do and what they actually do. There are also differences in the content of nurse education pharmaceutical care, making comparability between countries difficult [6–8].

International mobility of nurses in the European Union (EU) and worldwide is a growing phenomenon [9]. Several benefits have been described in the literature, including: balancing supply and demand within the workforce; health professionals trained abroad can fill gaps in care and shortages of nurses; greater cultural diversity; a lower average age to keep salary levels in check; and remittances to less affluent home countries [10, 11]. Healthcare professionals are permitted to practise anywhere in the EU’s single market and free movement zone. Given the differences in nurse education and practice across Europe, increasingly interconnected labour markets would benefit from more transparency in pharmaceutical care curricula. This also advocates for an international comparison of nurse educational programs.

The pharmaceutical care competency framework of Dijkstra et al. (2021) [12] describes several competencies that student nurses should master to be adequately prepared for clinical practice. To date, it is unclear to what extent pharmaceutical care competencies are integrated into current European curricula or whether nurses’ competencies are adequately addressed. Capturing the presence of domains and competencies related to pharmaceutical care in nurse education may lead to a better understanding of the extent to which curricula meet labour market expectations.

Therefore the aim of this study was to gain insight into the pharmaceutical care oriented content in nurse education at different educational levels, and students’ perceived readiness to provide nurse pharmaceutical care in clinical practice, with comparisons within and between countries.

## Methods

### Design

A quantitative cross-sectional study was conducted using a digital survey.

The study was part of a financed European project.

### Participants and setting

Students from 14 European countries participated in the study: Belgium, the Czech Republic, Germany, Greece, Hungary, Italy, the Netherlands, Norway, Portugal, the Republic of Northern Macedonia, Slovakia, Slovenia, Spain, and the United Kingdom (Wales and England). The countries were selected in an earlier phase of the overarching international Erasmus+ project (DeMoPhaC study) of which this study was the final part. Nursing schools that offered an educational program for students at levels 4, 5, 6, or 7 of the European Qualifications Framework (EQF) were approached to participate in the study [13]. Not all nurse educational levels existed in all countries. There are no level 5 students in the Netherlands, Norway, and Portugal, and level 4 students were included only in the Netherlands. In Wales, level 5 is not taught in the same higher education institutions as levels 6 and 7. Only nurse students in their final year of study were eligible, assuming that (almost) the entire curriculum had been covered.

### The digital survey

We used the CHERRIES statement for reporting web-based surveys to describe the development and use of the survey [14]. The survey was developed in English in consultation with one or two representatives of each participating country. All had expertise in nurse education. Questions about demographics, educational attainment oriented context, and the combination of studying and working in healthcare were followed by questions about pharmaceutical care in the nursing curriculum (Appendix 1). Students’ opinions about the presence of pharmaceutical care domains in the nursing curriculum and the extent to which they felt prepared to achieve pharmaceutical care-related competencies in practice were asked on a 5-point Likert scale ranging from 0 to 5 (0 = not at all; 5 = sufficiently). Domains of the NuPhaC-EU framework were used: management of medicines effects, medication adherence, patient medication self-management, patient education and information about medication, medication safety, and care coordination [5]. Then, the extent to which knowledge (10 questions), skills (10 questions), and attitudes (4 questions) about pharmaceutical care topics were present in the nursing curriculum was queried. This section was based on the pharmaceutical care competency framework developed by Dijkstra et al. (2021) [12]. Finally, students’ perceptions of opportunities to perform pharmaceutical care in previous clinical placements for educational purposes were assessed on a 4-point Likert scale ranging from 0 (strongly disagree) to 4 (strongly agree) [15].

### Validity and reliability/rigour

An education specialist from the Netherlands evaluated the questions (face validity) and advised on further validation and pilot testing. Subsequently, all English questions were presented to 14 experts (one from each participating country) from clinical practice, nurse education, and research who were asked to rate the relevance of all questions on a 4-point Likert scale ranging from not relevant, somewhat relevant, fairly relevant, to highly relevant [16]. The ‘Item Content Validity Index’ (I-CVI) corresponded to the number of experts who rated high or very high relevance divided by the number of participating experts (*n* = 14). Items with an I-CVI < of 0.80 (*n* = 8) were removed from the item pool, leaving 24 questions in this survey section. Finally, the English survey was translated into 13 languages in order to question all respondents in their local language. Translators were the representatives of each country, who were familiar with the study and therefore well positioned to take into account the cultural aspects and comprehensibility of the questions in relation to their country’s curricula and healthcare context, which resulted in ensuring that the intended meaning of all items was not lost. A pilot test in the local languages was conducted by six Belgian, Dutch, and Italian students to evaluate the comprehensibility and usability. No major adaptations were requested. The data from those six students were not used in the data analysis of this study.

### Data collection

Data collection took place between January and April 2021. In each country, at least five nurse educational programmes, per available level of education, were contacted and asked to encourage their students to participate. The online survey was available on a website developed for the purpose of this study. After completion of the questionnaire, students could benchmark their results nationally and internationally, and also within and between levels of education. A certificate of participation was available. The usability of the survey was further enhanced allowing students to pause the survey and restart it at a later time. In each country, local data collection strategies were considered to maximize data collection, taking into account restrictive measures and distance learning due to the COVID pandemic.

### Data analysis

Only students who completed at least half of the survey were included in the analysis. Data were analysed using IBM SPSS Statistics v28.0®. A two-sided level of 0.05 was used. Discontinuous data were described using frequency distributions; continuous data were described using median, minimum, and maximum. Normality of distributions was tested using absolute values of skewness and kurtosis or by calculating Z-scores as a function of (sub)sample size [17]. Differences between educational levels were examined. To assess the statistical significance of differences in the presence of pharmaceutical care in the curricula between the four educational levels, chi-square tests for categorical variables, one-way ANOVA tests for normally distributed scale variables, and Kruskal-Wallis tests for ordinal variables were used. Fisher exact tests were used for post-hoc analyses between two levels of education.

## Results

### General participant characteristics

A total of 112 institutions for nurse education were approached. Only students who completed at least half of the survey were included in the analysis. Therefore, 428 insufficiently completed questionnaires (less than 47% completed) were excluded from analyses. These students were mostly Italian (21%), Belgian (19%), Dutch (11%), Portuguese (8%), Slovakian (8%) or Spanish (8%). Age, work experience and working hours per week did not differ significantly between excluded and included students. Women had more incomplete questionnaires (18%) than men (14%, *p* < 0.001). Also, students, combining their studies with a job in healthcare quitted the survey more often too early (20%), then fulltime students (15%, *p* = 0.001).

Finally, 1807 students, predominantly female (78%), participated in the study. Students’ EQF level was 4 in 2%, 5 in 6%, 6 in 80%, and 7 in 12%. Median age was 23 (range 18–62) and median work experience in healthcare was 1 year (range 0–40). More than one quarter of the students were combining their studies with a job in healthcare, reporting a median of 36 (range 3–56) working hours per week. More detailed population characteristics are presented in Table 1.

**Table 1:** Population characteristics (*n* = 1807)

Participants	All (*n* = 1807)	Level 4 students (*n* = 26)	Level 5 students (*n* = 117)	Level 6 students (*n* = 1452)	Level 7 students (*n* = 212)
Characteristic					
**Country/State, %**					
Italy	66.2	0	0.9	74.8	51.4
Belgium	6.4	0	40.2	4.0	4.7
Spain	4.5	0	7.7	4.0	7.1
Republic of North Macedonia	3.2	0	12.8	1.7	9.0
Greece	3.3	0	5.1	3.3	2.4
The Netherlands	2.8	100	17.8	1.8	0
Portugal	2.6	0	0	2.4	5.7
Slovakia	2.2	0	0.9	2.1	3.3
Germany	1.9	0	16.2	0.9	0.9
UK - England	1.9	0	7.7	1.4	2.4
Slovenia	1.6	0	2.6	1.0	5.7
UK - Wales	1.1	0	0.9	1.2	0
Hungary	1.0	0	1.7	0.5	3.8
Czech Republic	0.7	0	3.4	0.3	1.4
Norway	0.6	0	0	0.5	2.4
**Gender, %**					
Male	21.6	11.5	9.4	22.9	20.8
Female	77.6	88.5	88.9	76.4	78.3
Other^a^	0.8	0	1.7	0.7	0.9
**Age** (years), median	23	22.5	27.0	22.0	30.0
(min-max)	(18–62)	(19–54)	(18–58)	(18–62)	(22–60)
**Experience in healthcare (years),** median	1	4	3	0	6
(min-max)	(0–40)	(1–34)	(0–38)	(0–36)	(0–40)
**Combining studies + job HC (yes),** %	28.9	92.3	35.0	20.1	78.8
Working hours healthcare/week, median	36	28	38	32	36
(min-max)^b^	(3–56)	(8–40)	(5–56)	(3–55)	(6–50)

^a^transgender male, transgender female, gender nonbinary, self-defined, prefer not to say or ‘other’; ^b^analysed in subsample of students combining studies with job in healthcare (*n* = 523)

### Pharmaceutical care in nursing curricula

#### Students perception about presence of pharmaceutical care content and readiness to apply competences

Up to 84% of all nursing students felt pharmaceutical care was insufficiently present in the current nursing curriculum. Similarly, an average of 14% (range 0–30%] felt sufficiently prepared to apply pharmaceutical care related competences in clinical practice. No difference was seen between levels of education related to presence of pharmaceutical care (*p* = 0.266) and feeling prepared for practice (*p* = 0.999), when questioning pharmaceutical care in general (no specific domain). Also, between the participating countries no difference existed for presence of pharmaceutical care (*p* = 0.129). Yet, opinions on the sufficiency of preparation of the curriculum did differ significantly: from no students feeling prepared in Hungary, to a maximum of 30% of the students in the Republic of North Macedonia (*p* < 0.001; see Table 2).

**Table 2:** Nurse students’ perceptions about the presence of knowledge, skills and attitudes related to pharmaceutical care topics in their curriculum in percentages (*n* = 1807)

Extent of presence in curriculum	Sufficiently present	Absent	Present but insufficiently	Unsure
Topics questioned				
**Knowledge about:**	%	%	%	%
Potential causes of drug related problems	70.4	3.6	24.3	1.8
Pharmacokinetics and pharmacodynamics	68.9	6.3	22.2	2.6
Which professional should be contacted to discuss treatment choices	68.4	7.1	18.9	5.6
The importance of sharing knowledge and medication-related information with patients and colleagues	68.2	6.4	22.3	3.1
Patient education about medication	66.6	5.8	24.3	3.3
Interventions that aim to prevent drug related problems & self-care	62.9	5.2	28.1	3.7
How to access information effectively to address drug related problems	61.2	7.3	27.9	3.7
How to obtain the best possible medication history and information on current medication regimen	55.8	8.5	29.9	5.8
National legislation^a^	41.7	16.9	35.1	6.3
The nurse independent/dependent prescribers’ formulary^a^	33.2	24.1	33.4	9.3
**Skills:**	%	%	%	%
Observing and recognizing therapeutic/adverse effects & DRPs	70.0	4.4	23.8	1.8
Empowering and involving the patient and/or family in PC	68.9	6.2	22.3	2.6
Undertaking safe storage, transportation and disposal of medicines for/with patients and/or patient advocates	64.7	8.6	22.6	4.1
Accessing medication-related information to address DRPs	63.4	7.4	24.8	4.3
Recognising needs & preferences of patient or family in self-management.	62.7	6.9	26.8	3.6
Applying interventions to optimise self-care	62.4	6.7	26.6	4.2
Obtaining timely, accurate, and thorough medication histories	59.7	8.3	28.3	3.7
Proposing and implementing interventions aiming to prevent DRPs	59.0	5.9	31.7	3.3
Proposing appropriate changes in medication therapy, including PRN	50.3	12.7	30.9	6.2
Prescribing and discontinuing medication listed in the nurse prescribers’ formulary or the independent prescribers’ formulary*	34.9	27.0	29.8	8.2
**Attitudes:**	%	%	%	%
Being able to verify patients’ understanding of information	79.2	3.1	14.7	3.0
Being able to respond to and respect patients’ preferences	76.5	3.6	16.2	3.7
Taking responsibility and a proactive attitude towards work needed to improve patients’ medication therapy	74.1	4.1	18.4	3.3
Having self-confidence to perform a task	71.5	5.9	19.2	3.4

*DRP* drug related problem, *PC* pharmaceutical care, *PRN* Pro Re Nata (= ‘if needed’ medication) ^a^competence reported as absent / insufficient / unsure by the majority of students (> 50%). Detailed percentages per level of education are presented in Fig. 2

During their education, nurse students gained practical experience through internships. Several opportunities to undertake pharmaceutical care within these clinical placements were observed: 80% reported an introduction to pharmaceutical care responsibilities and tasks in clinical practice, 70% had sufficient opportunities to undertake pharmaceutical care, 70% were satisfied with the supervision during undertaking pharmaceutical care, 75% saw an important role of nurse mentors in learning pharmaceutical care, and 65% received feedback from mentors on their development in pharmaceutical care. No statistical difference was found between the four EQF levels. (See Table 3).

**Table 3:** Pharmaceutical care in level 4 to 7 nurse curricula in 14 European countries – a) presence in the curriculum and b) preparation to achieve pharmaceutical care competences in clinical practice

Country / State	Students indicating PC as sufficiently present in their curriculum (%)	Students indicating their curriculum as sufficiently preparatory to achieve PC competences in practice (%)
Belgium (*n* = 112)	18.2	4.5
Czech Republic (*n* = 12)	18.2	16.7
Germany (*n* = 32)	6.3	6.3
Greece (*n* = 57)	19.6	12.3
Hungary (*n* = 17)	5.9	0.0
Italy (*n* = 1126)	15.8	14.1
Netherlands (*n* = 51)	14.3	17.6
Norway (n = 11)	18.2	9.1
Portugal (*n* = 47)	4.3	8.5
Republic of North Macedonia (*n* = 56)	14.0	30.4
Slovakia (*n* = 38)	10.5	10.5
Slovenia (*n* = 27)	4.2	3.7
Spain (*n* = 80)	20.0	11.3
UK - England (*n* = 34)	35.3	28.1
UK – Wales (*n* = 18)	11.1	15.8
*p*-value (difference between countries)	0.129	< 0.001

*PC* pharmaceutical care; p calculated with Chi Squared tests for the difference in % of students indicating PC as present in the curriculum between 14 countries (left column of table) and for the difference in % of students indicating the curriculum as sufficiently preparatory between 14 countries (right column of table)

#### Presence of pharmaceutical care related domains in nursing curricula

**Fig. 1:**
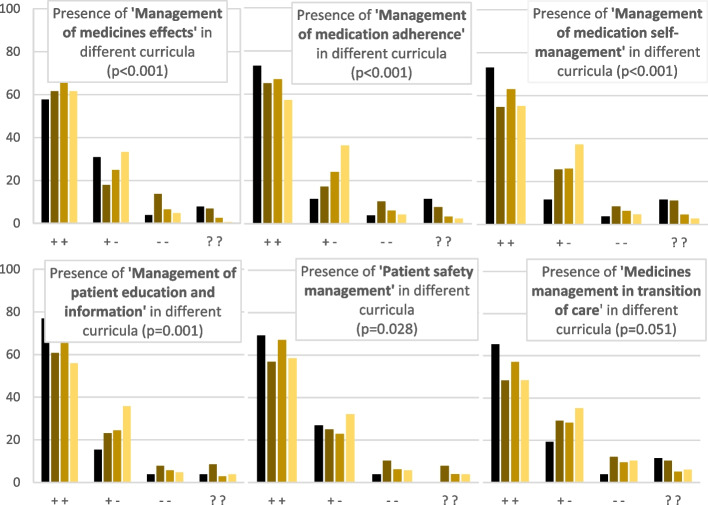
Reported presence of pharmaceutical care in nurse curricula, per educational level and pharmaceutical care domain. Legend: Bar colours:  level 4 (*n* = 26)  level 5 (*n* = 120)  level 6 (*n* = 1485)  level 7 (*n* = 217). X-axis symbols: + + = sufficiently present + − = present, but insufficiently - - = absent?? = unsure. *p*-values calculated with chi squared tests

When looking at six specific pharmaceutical care domains in which nurses can have responsibilities, we found more than half of all students considered each domain sufficiently present in their curriculum: management of medicines effects (65%), management of medicines adherence (66%), management of medication self-management (62%), patient education (66%), patient safety management (66%) and transition of care coordination (56%). In the perceptions of students, in level 7 curricula the different domains were reported more frequently as ‘present, but insufficiently’ compared to the other levels. More level 5 students reported these domains to be absent than level 4, 6 and 7 students (*p* < 0.05 for all domains except for transition of care). Figure 1 shows the six domains per educational level. In Appendix 2 these data are presented for the 14 European countries.

#### Presence of pharmaceutical care related competences in nursing curricula

When exploring the presence of topics, related to knowledge, skills and attitudes of nurses in pharmaceutical care, most were experienced as sufficiently present by the majority (range 50.3–79.2%) of all nurse students. Knowledge about national legislation, knowledge about the nurse prescribers’ formulary and skills around prescribing and discontinuing medication were perceived as least present. Respectively 58, 67 and 65% of the students reported these topics as absent, insufficiently present or uncertainly present (Table 4).

**Table 4:** Nurse students’ perceptions about opportunities to undertake pharmaceutical care within their previous clinical placements, split up for 4 levels of nurse education

Opportunity to undertake PC within previous clinical placements	All students (n = 1807)	Level 4 students (n = 26)	Level 5 students (n = 117)	Level 6 students (n = 1452)	Level 7 students (n = 212)	p
Student has been introduced to responsibilities and tasks relating PC in clinical practice (yes, %)	80.0	73.1	76.9	80.1	81.5	0.620
Student has had sufficient opportunity to undertake PC in clinical practice (yes, %)	69.7	76.9	70.9	69.0	73.1	0.520
Student is satisfied with the supervision I have received when undertaking PC in clinical practice (yes, %)	69.9	88.5	67.5	70.0	67.9	0.173
Nurse mentors have had an important role in how the student has learned PC in practice (yes, %)	74.8	84.6	80.3	74.4	73.6	0.317
Student has received mentors’ feedback on their development in PC (yes, %)	65.5	80.8	72.6	64.7	65.1	0.123

*PC* Pharmaceutical care. p calculated with Chi-Squared tests

**Fig. 2:**
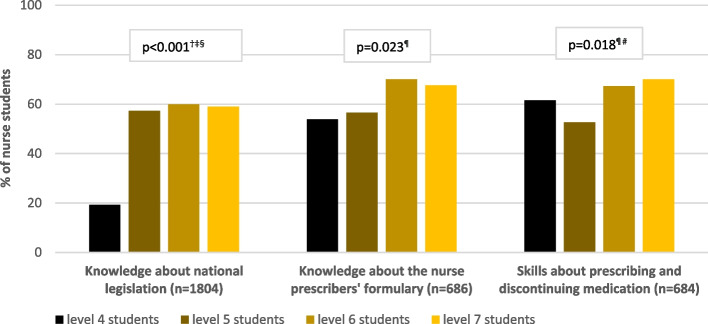
Pharmaceutical care related knowledge and skills reported as absent, insufficiently present or unsure in level 4, 5, 6 and 7 nurse curricula by the majority of the respondents*.* ^†^*p* < 0.05 between level 4–5; ^‡^*p* < 0.05 between level 4–6; ^§^*p* < 0.05 between level 4–7; ^¶^*p* < 0.05 between level 5–6; ^#^*p* < 0.05 between level 5–7. *p*-values calculated with Chi-Squared and Fisher Exact tests

Significantly more level 5, 6 and 7 students (respectively 57, 60, 59%) missed national legislation topics in their pharmaceutical care curriculum than level 4 students did (19%; *p* < 0.001 for all three comparisons). Also, less level 5 students reported knowledge about the nurse prescribers’ formulary (57%) and skills about prescribing and discontinuing medication (53%) than level 6 students (respectively 70%, *p* = 0.007 and 67%, *p* = 0.004; Fig. 2).

For 22 topics more Italian students reported pharmaceutical care as sufficiently present in the curriculum compared to non-Italian students (all *p* < 0.05). To provide a broad picture of the European situation, both overall data (Table 4) as well as a detailed overview of Italian versus non-Italian curricula (Table 5) are presented.

**Table 5:** Students nurses’ perceptions about the presence of knowledge, skills and attitudes related to pharmaceutical care topics in their curriculum in percentages, split up for Italian students and non-Italian students *(n* = 1807)

	Italian student nurses				Other European students				*p*-value (Italy vs non-Italy)
	Sufficiently present	Absent	Present but insufficient	Unsure	Sufficiently present	Absent	Present but insufficient	Unsure	
**Knowledge about:**									
Potential causes of drug related problems	74.8	4.8	18.6	1.8	57.2	9.2	29.3	4.3	< 0.001
Pharmacokinetics and pharmacodynamics	42.1	17.3	34.8	5.8	41.1	15.9	35.6	7.4	0.510
Which healthcare professional should be contacted to discuss treatment choices/changes	76.7	2.9	19.3	1.1	58.1	4.8	34.0	3.1	< 0.001
Importance of sharing medication-related information with patients/colleagues	66.2	4.1	26.4	3.3	56.4	7.4	31.6	4.6	< 0.001
Patient education about medication	73.7	5.4	16.3	4.6	57.7	10.5	24.2	7.6	< 0.001
Interventions that aim to prevent drug related problems & self-care	71.3	4.9	20.4	3.4	57.4	7.4	31.9	3.3	< 0.001
How to access information effectively to address drug related problems	60.7	6.9	27.4	5.0	46.2	11.8	34.8	7.2	< 0.001
How to obtain the best possible medication history/information on current regimen	39.2	10.3	34.0	16.5	32.3	26.3	33.3	8.1	< 0.001
National legislation	67.4	5.4	23.8	3.4	49.0	11.0	35.7	4.3	< 0.001
The nurse independent/dependent prescribers’ formulary	73.4	4.9	18.9	2.8	58.0	9.5	28.9	3.6	< 0.001
**Skills:**									
Observing and recognizing therapeutic/adverse effects & drug related problems	74.2	4.0	20.2	1.6	61.9	5.1	30.9	2.1	< 0.001
Empowering and involving the patient and/or family in pharmaceutical care	63.6	5.1	27.7	3.6	50.1	7.6	39.5	2.8	< 0.001
Undertaking safe medicines storage, transportation & disposal for/with patients	63.9	6.7	25.6	3.8	59.6	6.7	28.6	5.1	0.231
Accessing medication-related information to address drug related problems	64.8	6.7	24.5	4.0	49.8	11.3	35.8	3.1	< 0.001
Recognising the needs and preferences of the patient and/or family in self-management	54.8	11.0	28.1	6.1	41.3	16.0	36.4	6.3	< 0.001
Applying interventions to optimise self-care	67.1	5.6	23.8	3.4	53.8	9.6	32.6	4.0	< 0.001
Obtaining timely, accurate, and thorough medication histories	74.4	4.4	19.0	2.2	58.2	9.7	28.6	3.5	< 0.001
Proposing and implementing interventions aiming to prevent drug related problems	67.6	7.3	21.2	4.0	59.1	11.2	25.6	4.1	< 0.001
Proposing appropriate changes in medication therapy, including PRN	47.9	16.7	26.0	9.4	32.9	28.7	30.4	8.0	0.014
Prescribing + discontinuing medication listed in nurse/independent prescribers’ formulary	69.6	5.4	21.2	3.8	51.4	11.4	31.9	5.3	< 0.001
**Attitudes:**									
Being able to verify patients’ understanding of information	76.2	5.3	15.8	2.7	62.1	7.1	25.9	4.9	< 0.001
Being able to respond to and respect patients’ preferences	81.4	3.5	12.9	2.2	59.7	5.3	29.4	5.6	< 0.001
Taking responsibility/proactive attitude to improve medication therapy	79.4	3.8	14.4	2.4	70.6	3.3	19.9	6.2	< 0.001
Having self-confidence to perform a task	82.5	3.3	12.4	1.8	72.9	2.6	19.2	5.3	< 0.001

## Discussion

The purpose of this study was to assess the presence of pharmaceutical care related courses in nursing curricula and to gain insight into pharmaceutical care in these curricula at different levels of education. Pharmaceutical care was considered inadequate in current nursing curriculum by more than three-quarters of all students, and only 14% felt adequately prepared to achieve pharmaceutical care related competencies in clinical practice. These alarming results were found when nursing students were surveyed about pharmaceutical care by nurses, defined as ‘nurses’ contribution to the care of individuals in terms of optimizing medication use and improving health outcomes’. When we looked more closely at the presence of pharmaceutical care by examining nursing students’ opinions on various pharmaceutical care domains, we found moderate results that nevertheless revealed a lack of pharmaceutical care in current curricula. Our data showed that only 56 to 66% of all students considered the various domains of pharmaceutical care to be sufficiently present. This means that at least one-third of our sample missed courses that could optimally prepare them to assume responsibilities in interprofessional pharmaceutical care. Finally, our results indicate that a significant proportion of students perceived the presence of knowledge and skills related to pharmaceutical care topics in their curriculum as insufficient. Level 5 curricula lacked more pharmaceutical care areas than the other levels. Similarly, knowledge about the nurse prescribers’ formulary and medication discontinuation skills were less present in Level 5 than in Level 6.

### Implications for education, policy, and future research

The vast majority of all patients are treated with medicines. Pharmaceutical care with all its domains is therefore of great importance in clinical practice. The prominent role of nurses in pharmaceutical care has already been widely demonstrated [1, 18, 19]. Consequently, nursing students should be maximally qualified to perform the wide range of tasks in pharmaceutical care. However, our survey found that final-year students are inadequately prepared for the expanded role that awaits them at pharmaceutical care. These results confirm the findings in earlier studies about the need for greater integration of pharmaceutical care into existing curricula [20, 21]. Dilles et al. (2011) found that pharmacology knowledge and computational skills of nursing students were limited just prior to graduation [21]. As in our study, students did not feel confident using pharmaceutical care in practice. The fact that researchers [1, 20] expressed concern about the discrepancy between education and practice regarding pharmaceutical care more than 20 years ago should be a wake-up call to policymakers. There is an urgent need to address current deficiencies in nurse practitioner curricula. Pharmaceutical care is a complex process that involves multiple management and treatment decisions. Compared to pharmacy curricula, specific courses strictly dedicated to pharmaceutical care and named as such are not part of the nurse curricula. As a recommendation for policy makers or nurse educators, it should be clear which ECTS are dedicated to pharmaceutical care in dedicated courses, or integrated in other courses. Several subsets of pharmaceutical care are considered deficient in current nursing education. This raises concerns about the quality of education for current nursing students [22]. The lack of curriculum consistency in relation to pharmaceutical care should be addressed to increase the coherence of nursing education programs and better prepare students to transition into clinical practice. In particular, if students are not adequately prepared for pharmaceutical care in practice, this can lead to an increased risk of (nursing) errors and medication errors [22]. Therefore, in anticipation of greater incorporation of pharmaceutical care knowledge, skills, and attitudes into nursing curricula, we ask registered nurses and other healthcare professionals to be aware of the potential limitations of final-year nursing students and newly graduated nurses in practice pharmaceutical care.

Our study took part 2 years after the start of the COVID pandemic, meaning that most of the education of the final-year participants took place during the pandemic. Pre-COVID research already suggested a need for more integration of pharmaceutical care into existing curricula. Our results are in line with these findings and add new insights, specifying the domains and an international perspective. It should be considered, however, that there might have been a negative impact of the pandemic on students’ learning. The pandemic has shown a negative influence on competency areas and nursing practice readiness [23].

We are aware that budgets in both clinical practice and nursing education are under pressure in most European countries [24]. Savings in healthcare practice are leading to major changes, such as shorter and shorter hospital stays, which in turn pose a major challenge to healthcare workers and patients [25]. Strategies need to be developed to help healthcare workers manage medications safely through standardized training. To address the existing variance of pharmaceutical care in nurse education, we recommend revising current curricula based on the NUPHAC-EU framework and the pharmaceutical care competency framework [5, 12]. A course exclusively devoted to pharmaceutical care (and also so-called) would be helpful. We recognize that implementation of both frameworks will take time and there are significant costs to consider. However, a cost-benefit analysis should always keep in mind that the benefits of well-prepared students and nurses providing safe health care with better patient outcomes is actually priceless. Therefore, we recommend that European governments provide incentives to educational institutions to implement the framework we propose. We strongly advise using funds from the European Union Health Program or research funds for health and well-being, rather than funding them from tax dollars levied on employers and the public.

### Strengths and limitations

Comparing different education systems is challenging because the terms are not always used in the same way in different countries. Back in 2014, Lahtinen et al. highlighted the lack of accessible and reliable information about education programs in nursing [6]. Also different cultural and legislative contexts, determining nurses’ role in EU countries, have been observed previously [19]. Our findings are a step toward more transparency in nurse education curricula related to pharmaceutical care.

The greatest strength of our study was the overview of the perceived pharmaceutical care gaps in the current curricula for nurses: six pharmaceutical care domains, as defined by De Baetselier et al. (2021) [5], were present in the educational programs, but at least one third of the students reported that they were insufficiently present or absent.

This internet survey had limitations. The inclusion or exclusion of educational institutions and students was determined by whether they agreed or declined to participate in the study. This self-selected sample with an unknown response rate might have led to a distortion of the results due to only the most motivated student nurses participating. The survey was tested by six students from only 3 countries, resulting in an incomplete pilot test for all countries. Nevertheless, we can guarantee that the translation by representatives from each country took into account cultural aspects and the comprehensibility of the questions in relation to curricula and health care context in the country. Our study surveyed a large sample of European nursing students, two-thirds of whom were Italian. The reason for this preponderance of Italians was the possibility of offering ECTS credit to every Italian student. This greatly aided data collection in Italy, but seriously skewed our ‘European’ level 6 and 7 data. However, we believe that by presenting the available data in a transparent way, the percentages reported can be interpreted in a European context. We have shown data for both individual countries as well as comparisons between Italy and the rest of Europe. Nevertheless, we recommend expanding the sample in non-Italian countries, because of the disappointing response rate and non-completion rate, to allow for country-level recommendations. Further research can learn from the Italian strategy to include students. In addition, technical problems encountered in some countries should be addressed to better ensure usability and prevent premature survey abandonment.

To evaluate the nurse practitioner curriculum, we surveyed students rather than teachers. Ideally, we would have preferred to include a representative (e.g., coordinator, director, ...) in each school to complete a questionnaire about the curriculum. However, we knew from previous experience in our research group that representatives in nursing schools are not always fully or currently informed about every aspect of the curriculum. Therefore, we had to consider that surveying this audience would have resulted in a significant number of missing or outdated responses. In addition, some schools of nursing are a frequently surveyed group in research (e.g., students), which could have resulted in a high non-response rate. To prevent this, we surveyed final-year nursing students about their curriculum, although we are aware that students’ self-reported data could also be biased.

## Conclusion

Although several pharmaceutical care-related courses are present in current level 4 to 7 nurse curricula, the embedding of pharmaceutical care should be expanded. More areas of pharmaceutical care were missing from the level 5 curricula than from the other levels. Overall, too many students indicated that pharmaceutical care responsibilities for nurses in interprofessional pharmaceutical care were inadequate or missing and perceived inadequate preparation for learning pharmaceutical care competencies in real-world practice. Existing gaps in pharmaceutical care should be addressed to provide more thoroughly prepared nurses to the labour market.

### Supplementary Information

**Additional file 1.**

**Additional file 2.**

## Data Availability

All data generated or analysed during this study are included in this published article and its supplementary information files.

## Abbreviations

EU: European Union
EQF: European Qualifications Framework
I-CVI: Item Content Validity Index
PC: Pharmaceutical Care
