# Supplementary material for: Cross-sectional evaluation of pharmaceutical care competences in nurse education: how well do curricula prepare students of different educational levels?
Source: BMC Nurs. 2024 Feb 6;23:96. doi: 10.1186/s12912-023-01646-6 (PMC10845807; doi:10.1186/s12912-023-01646-6)
Supplement: Supplementary file 1 — Additional file 1. [file 12912_2023_1646_MOESM1_ESM.pdf]

Appendix S1: Survey

Demographical questions

Q1 In which country are you currently studying?

0_1 Belgium (België)
0_2 Czech Republic (Česká republika)
0_3 Germany (Deutschland)
0_4 Greece (Ελλάδα)
0_5 Hungary (Magyarország)
0_6 Italy (Italia)
0_7 The Netherlands (Nederland)
0_8 Norway (Norge)
0_9 Portugal
0_{10} Republic of North Macedonia (Република Северна Македонија)
0_{11} Slovakia (Slovensko)
0_{12} Slovenia (Slovenija)
0_{13} Spain (España)
0_{14} United Kingdom (England)
0_{15} United Kingdom (Wales)

Q2 Level of education?

Q3 Do you identify as:

0_1 Male
0_2 Female
0_3 Other
0_4 Prefer not to say

Q4 What is your age? …. Years

Q5 Are you combining your studies with a job in healthcare? (Either paid or voluntary)

0_0 No → question Q6 will not be showed / go to Q7
0_1 Yes

Q6 How many hours per week are you currently working in healthcare? …. Hours

Q7 How many years of work experience do you have in healthcare? …. Years

Questions about pharmaceutical care in your nursing curriculum

When we talk about pharmaceutical care by nurses, we want you to think about nurses' contribution to the care of individuals in order to optimize medicines use and improve health outcomes.

A/ NURSING CURRICULUM – MORE GENERAL QUESTIONS

Q8 Is pharmaceutical care sufficiently present in the current nursing curriculum? Indicate your opinion on a scale from 0 to 5 (0 = not at all, 5 = sufficiently present).

0	1	2	3	4	5

Q9 In previous research, we identified several responsibilities for nurses in interprofessional pharmaceutical care. Indicate for each of the following responsibilities whether they were absent in your nursing curriculum, present but insufficient, sufficiently present or that you don't know.

	absent	present but insufficient	sufficient	Unsure
**Q9.1** Management of therapeutic and adverse effects of medicines (monitoring, assessing needs, identification of drug related problems, follow-up)	O_0	O_1	O_2	O_3
**Q9.2** Management of medication adherence (monitoring, assessing needs, identification, motivational interviewing, follow-up)	O_0	O_1	O_2	O_3
**Q9.3** Management of medication self-management (empowerment, self-care support, assessing needs)	O_0	O_1	O_2	O_3
**Q9.4** Management of patient education and information (communication, discussion, counselling, coaching, training, advice)	O_0	O_1	O_2	O_3
**Q9.5** Patient safety management (medication error reporting, follow-up)	O_0	O_1	O_2	O_3
**Q9.6** Medicines management in discharge planning and transition of care	O_0	O_1	O_2	O_3

Q10 Did the current nursing curriculum sufficiently prepare you to achieve pharmaceutical care related competences in clinical practice? Indicate your opinion on a scale from 0 to 5 (0 = not at all, 5 = sufficiently prepared).

0	1	2	3	4	5

Q11 The following items question your perceptions about opportunities to undertake pharmaceutical care within your previous clinical placements. Please indicate for each of the following statements whether you strongly disagree, disagree, agree or strongly agree.

Statement	Strongly disagree	Disagree	Agree	Strongly agree
**Q11.1** I have been introduced to responsibilities and tasks relating pharmaceutical care in clinical practice	O_0	O_1	O_2	O_3
**Q11.2** I have had sufficient opportunity to undertake pharmaceutical care in clinical practice	O_0	O_1	O_2	O_3
**Q11.3** I am satisfied with the supervision I have received when undertaking pharmaceutical care in clinical practice	O_0	O_1	O_2	O_3
**Q11.4** Nurse mentors have had an important role in how I have learned pharmaceutical care in practice	O_0	O_1	O_2	O_3

**Q11.5** I have received feedback from mentors on my development in pharmaceutical care	O_0	O_1	O_2	O_3

B/ NURSING CURRICULUM – KNOWLEDGE

Indicate for each of the following items to what extent knowledge related to these topics was present in your nursing curriculum.

Knowledge about:	Absent	Present but insufficient	Sufficient	Unsure
**Q12.1** Pharmacokinetics and pharmacodynamics	O_0	O_1	O_2	O_3
**Q12.2** National legislation	O_0	O_1	O_2	O_3
**Q12.3** Potential causes of drug related problems	O_0	O_1	O_2	O_3
**Q12.4** Interventions that aim to prevent drug related problems and self-care	O_0	O_1	O_2	O_3
**Q12.5** Which healthcare professional should be contacted to discuss treatment choices/changes	O_0	O_1	O_2	O_3
**Q12.6** Patient education about medication	O_0	O_1	O_2	O_3
**Q12.7** How to obtain the best possible medication history and information on current medication regimen	O_0	O_1	O_2	O_3
**Q12.8** The nurse independent/dependent prescribers' formulary	O_0	O_1	O_2	O_3
**Q12.9** How to access medication-related information effectively to address drug related problems	O_0	O_1	O_2	O_3
**Q12.10** The importance of sharing knowledge and medication-related information with patients and colleagues	O_0	O_1	O_2	O_3

C/ NURSING CURRICULUM – SKILLS

For the following items indicate to what extent the relevant skills were present in your nursing curriculum.

Skills about:	Absent	Present but insufficient	Sufficient	Unsure
**Q13.1** Observing and recognizing therapeutic effects, adverse effects and drug-related problems	O_0	O_1	O_2	O_3
**Q13.2** Proposing and implementing interventions aiming to prevent drug-related problems	O_0	O_1	O_2	O_3
**Q13.3** Applying interventions to optimise self-care	O_0	O_1	O_2	O_3
**Q13.4** Obtaining timely, accurate, and thorough medication histories	O_0	O_1	O_2	O_3
**Q13.5** Proposing appropriate changes in medication therapy, including PRN	O_0	O_1	O_2	O_3
**Q13.6** Recognising the needs and preferences of the patient and/or family in self-management.	O_0	O_1	O_2	O_3
**Q13.7** Empowering and involving the patient and/or family in pharmaceutical care	O_0	O_1	O_2	O_3
**Q13.8** Undertaking safe storage, transportation and disposal of medicines for/with patients and/or patient advocates	O_0	O_1	O_2	O_3

**Q13.9** Prescribing and discontinuing medication listed in the nurse prescribers’ formulary or the independent prescribers' formulary	O_0	O_1	O_2	O_3
**Q13.10** Accessing medication-related information to address drug related problems	O_0	O_1	O_2	O_3

D/ NURSING CURRICULUM – ATTITUDES

Indicate for each item to what extent education of attitudes about these topics was present in your nursing curriculum.

Attitude about:	Absent	Present but insufficient	Sufficient	Unsure
**Q14.1** Having self-confidence to perform a task	O_0	O_1	O_2	O_3
**Q14.2** Taking responsibility and a proactive attitude towards work needed to improve patients' medication therapy	O_0	O_1	O_2	O_3
**Q14.3** Being able to respond to and respect patients' preferences	O_0	O_1	O_2	O_3
**Q14.4** Being able to verify patients’ understanding of education / information	O_0	O_1	O_2	O_3
