# Supplementary material for: Cross-sectional evaluation of pharmaceutical care competences in nurse education: how well do curricula prepare students of different educational levels?
Source: BMC Nurs. 2024 Feb 6;23:96. doi: 10.1186/s12912-023-01646-6 (PMC10845807; doi:10.1186/s12912-023-01646-6)
Supplement: Supplementary file 2 — Additional file 2. [file 12912_2023_1646_MOESM2_ESM.pdf]

Appendix S2: The reported presence of pharmaceutical care in nurse curricula, split up for 14 countries, three educational levels and six pharmaceutical care domains (n = 1848)

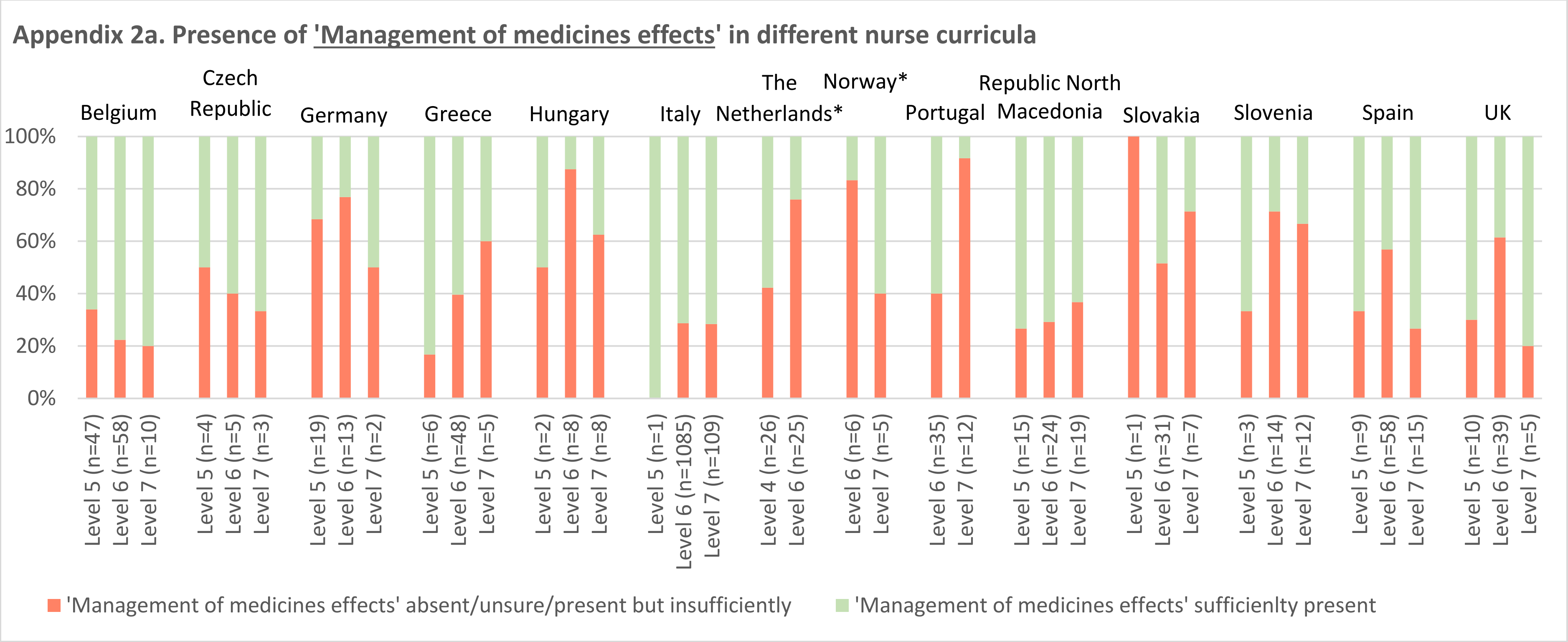

* p<0.05 in the Netherlands and Norway; p calculated with Chi Squared tests for the difference in reported presence of management of medicines effects between three educational programmes. Fisher Exact was used, if only two levels of education were available.

Appendix 2b. Presence of 'Management of medicines adherence' in different nurse curricula

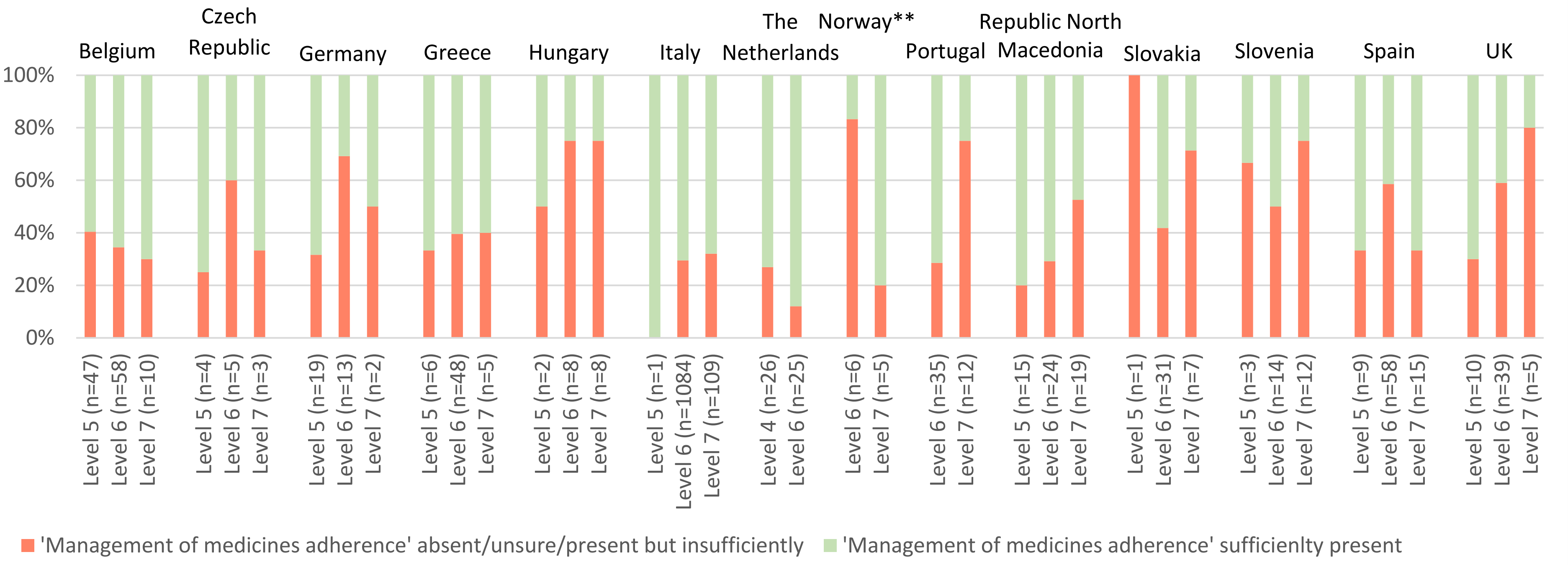

* p<0.05 in Norway; p calculated with Chi Squared tests for the difference in reported presence of management of medicines adherence between three educational programmes. Fisher Exact was used, if only two levels of education were available.

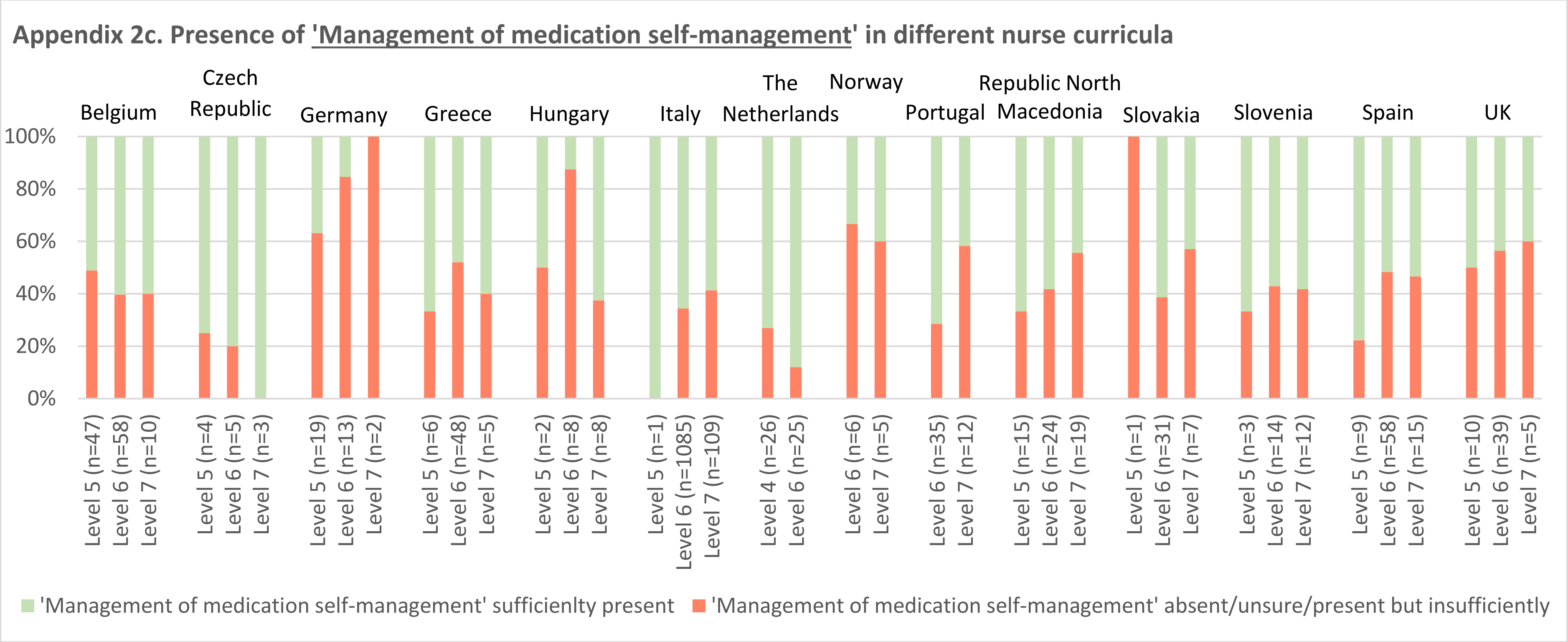

All p>0.05; p calculated with Chi Squared tests for the difference in reported presence of management of medicines adherence between three educational programmes. Fisher Exact was used, if only two levels of education were available.

Appendix 2d. Presence of '<u>patient education & information about medication</u>' in different nurse curricula

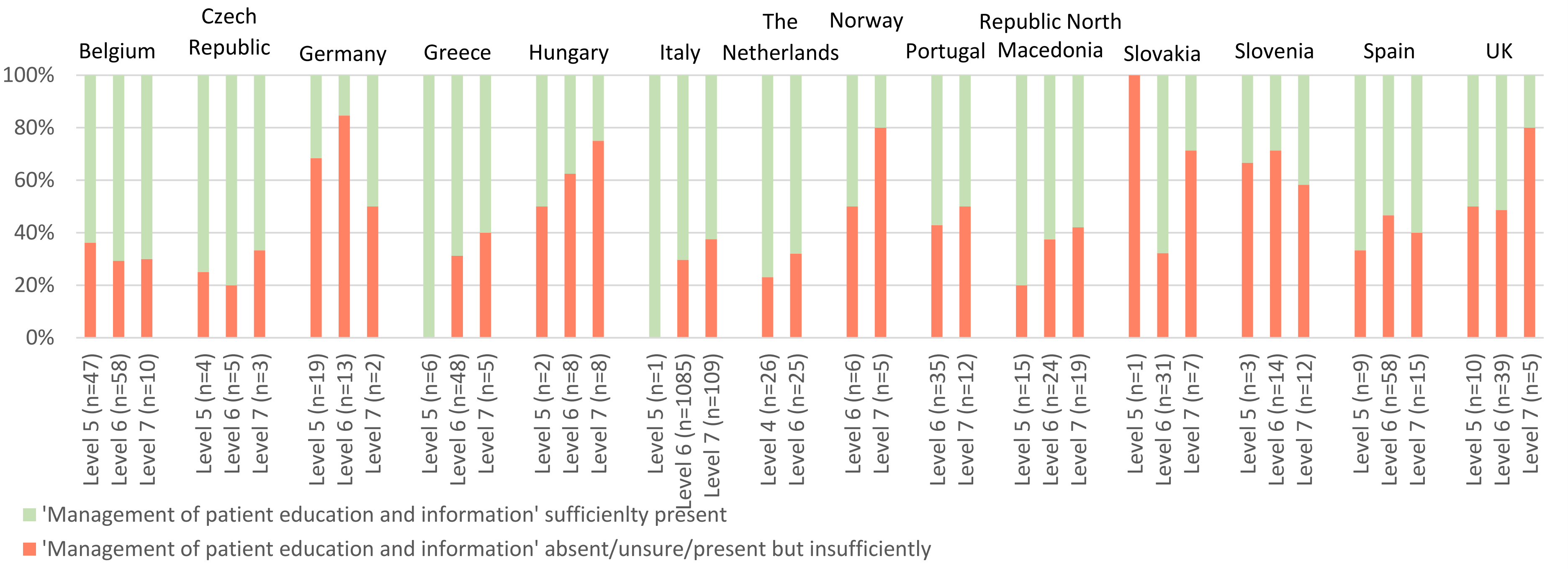

All p>0.05; p calculated with Chi Squared tests for the difference in reported presence of patient education and information between three educational programmes. Fisher Exact was used, if only two levels of education were available.

Appendix 2e. Presence of '<u>patient safety management</u>' in different nurse curricula

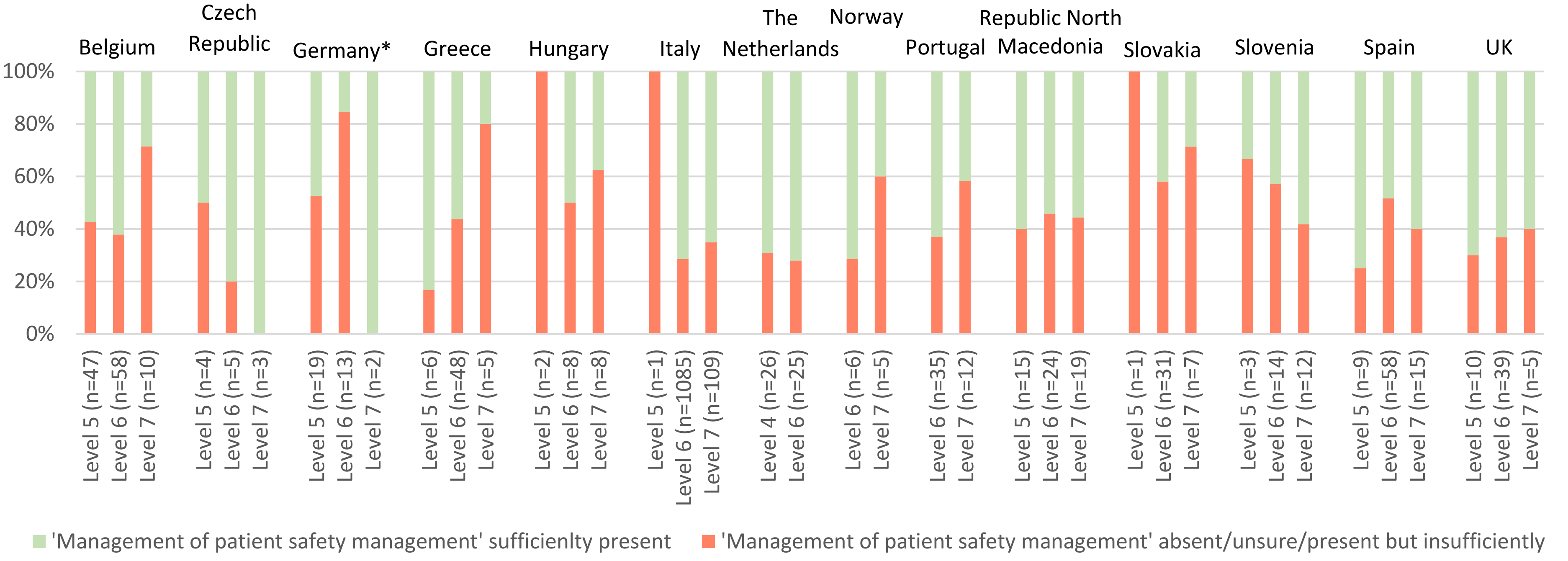

* p<0.05 in Germany; p calculated with Chi Squared tests for the difference in reported presence of management of medicines adherence between three educational programmes. Fisher Exact was used, if only two levels of education were available.

Appendix 2f. Presence of ''medicines management in transition of care' in different nurse curricula

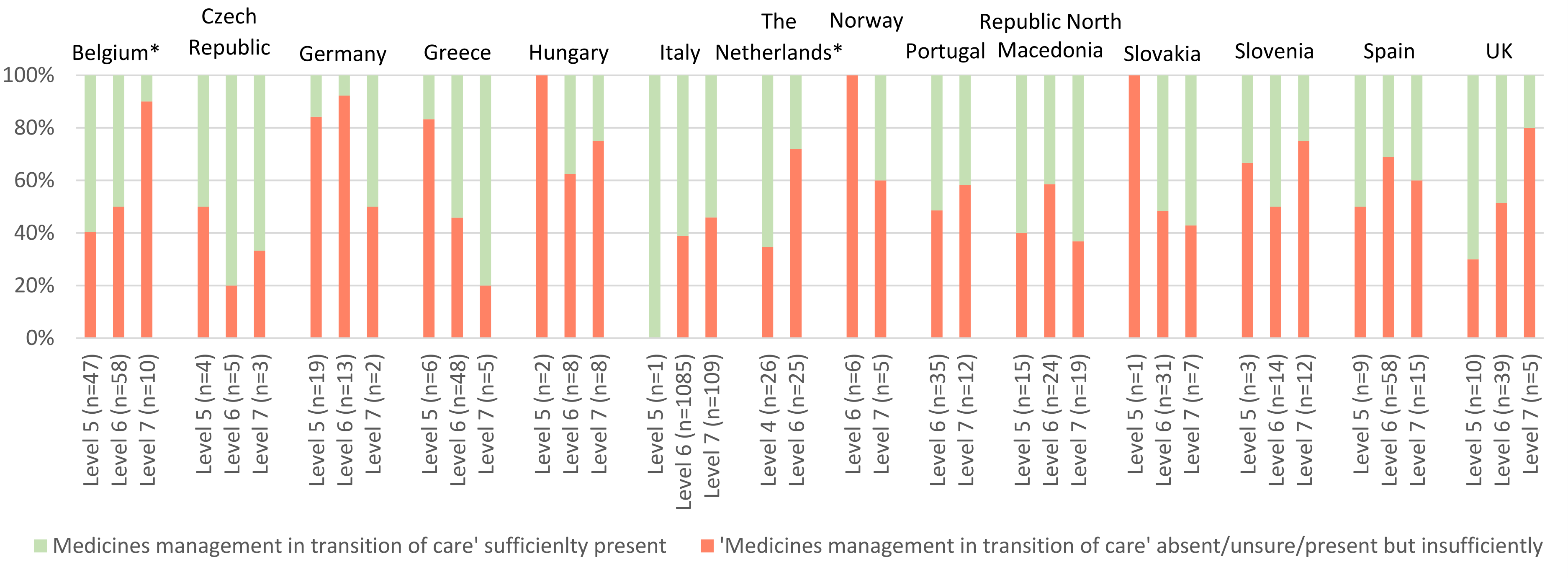

* p<0.05 in Belgium and the Netherlands; p calculated with Chi Squared tests for the difference in reported presence of management of medicines adherence between three educational programmes. Fisher Exact was used, if only two levels of education were available.
